# Psychotropic medication use among patients with a traumatic brain injury treated in the intensive care unit: a multi-centre observational study

**DOI:** 10.1007/s00701-021-04956-3

**Published:** 2021-08-11

**Authors:** Juho Vehviläinen, Markus B. Skrifvars, Matti Reinikainen, Stepani Bendel, Ivan Marinkovic, Tero Ala-Kokko, Sanna Hoppu, Ruut Laitio, Jari Siironen, Rahul Raj

**Affiliations:** 1 Department of Neurosurgery, Helsinki University Hospital and University of Helsinki, Topeliuksenkatu 5, P.B. 266, 00029 HUS Helsinki, Finland; 2 Department of Emergency Care and Services, University of Helsinki and Helsinki University Hospital, Helsinki, Finland; 3 Department of Intensive Care, Kuopio University Hospital & University of Eastern Finland, Kuopio, Finland; 4 Department of Neurology, Helsinki University Hospital and University of Helsinki, Helsinki, Finland; 5 Department of Intensive Care, Oulu University Hospital & University of Oulu, Oulu, Finland; 6 Department of Intensive Care and Emergency Medicine Services, Tampere University Hospital & University of Tampere, Tampere, Finland; 7 Department of Intensive Care, Turku University Hospital & University of Turku, Turku, Finland

**Keywords:** Traumatic brain injury, Psychiatric sequelae, Intensive care, Psychotropic medication

## Abstract

**Background:**

Psychiatric sequelae after traumatic brain injury (TBI) are common and may impede recovery. We aimed to assess the occurrence and risk factors of post-injury psychotropic medication use in intensive care unit (ICU)-treated patients with TBI and its association with late mortality.

**Methods:**

We conducted a retrospective multi-centre observational study using the Finnish Intensive Care Consortium database. We included adult TBI patients admitted in four university hospital ICUs during 2003–2013 that were alive at 1 year after injury. Patients were followed-up until end of 2016. We obtained data regarding psychotropic medication use through the national drug reimbursement database. We used multivariable logistic regression models to assess the association between TBI severity, treatment-related variables and the odds of psychotropic medication use and its association with late all-cause mortality (more than 1 year after TBI).

**Results:**

Of 3061 patients, 2305 (75%) were alive at 1 year. Of these, 400 (17%) became new psychotropic medication users. The most common medication types were antidepressants (61%), antipsychotics (35%) and anxiolytics (26%). A higher Glasgow Coma Scale (GCS) score was associated with lower odds (OR 0.93, 95% CI 0.90–0.96) and a diffuse injury with midline shift was associated with higher odds (OR 3.4, 95% CI 1.3–9.0) of new psychotropic medication use. After adjusting for injury severity, new psychotropic medication use was associated with increased odds of late mortality (OR 1.19, 95% CI 1.19–2.17, median follow-up time 6.4 years).

**Conclusions:**

Psychotropic medication use is common in TBI survivors. Higher TBI severity is associated with increased odds of psychotropic medication use. New use of psychotropic medications after TBI was associated with increased odds of late mortality. Our results highlight the need for early identification of potential psychiatric sequelae and psychiatric evaluation in TBI survivors.

**Supplementary Information:**

The online version contains supplementary material available at 10.1007/s00701-021-04956-3.

## Introduction

Survivors of traumatic brain injury (TBI) and intensive care unit (ICU) treatment suffer from long-term neurological, psychiatric and social burden, in spite of favourable functional recovery [10, 22, 26, 37–39]. Patients with TBI may suffer from various psychiatric problems, such as depression, bipolar affective disorder and anxiety disorders, which are likely to impede recovery [12, 17, 29, 33, 37]. In general, patients suffering from any trauma seem to be susceptible to psychiatric problems following ICU admission [35]. In addition, a significant proportion of patients with TBI have psychiatric comorbidities prior to injury, the three most common being substance use disorder, major depressive disorder and anxiety [39]. Indeed, psychiatric comorbidities are among the strongest predictors of late mortality (death 1 year or later after discharge) among patients with TBI [4].

In recent years, there has been an increase in the use of psychotropic medications among the general population in many high-income countries. In Finland, the prevalence of psychotropic medication use was 18% in the adult population in 2013 [40]. Of all psychotropic medications, antidepressants is the most common medication used among TBI patients [2]. In Europe between the years 2000 and 2010, the prevalence of antidepressant use increased from 3.7 to 7.2% [24]. In parallel, the global incidence of TBI is increasing [14]. Thus, given the negative impact of psychiatric sequelae on recovery of patients with TBI, there is a need to understand the complex interplay between pre- and post-injury psychotropic medication use and TBI care.

Accordingly, we designed the current study to gain data on the prevalence of psychotropic medication use, as a proxy of psychiatric disorders, among patients with TBI treated in the ICU. We set out to study whether certain injury types, or any particular clinical course, would associate with the need for psychotropic medications among TBI survivors. We further studied whether new or previous use of psychotropic medications was associated with in increased risk for late all-cause mortality in 1-year survivors. We hypothesized that (1) the use of psychotropic medications would be common both pre- and post-TBI, (2) that higher TBI severity would increase the use of psychotropic medication in survivors and (3) that TBI survivors using psychotropic medications would have an increased risk for late mortality.

## Methods and materials

The ethics committee of Helsinki University Hospital (194/13/03/14 §97), the Finnish National Institute for Health and Welfare (THL/713/5.05.01/2014 and THL/1298/5.05.00/2019), Statistics Finland (TK-53–1047-14), the Social Insurance Institution of Finland (Kela 23/522/2018), the Office of the Data Protection Ombudsman (Dnro 2713/402/2016 28.10.16) and all the participating university hospitals’ research committees approved this study. The study adhered to the Strengthening the Reporting of Observational studies in Epidemiology (STROBE) guidelines. We retrieved data on mortality from the Finnish population register on December 31, 2016 (available for all Finnish residents).

### Study design and population

We performed a multi-centre retrospective observational study using data that were prospectively collected to the Finnish Intensive Care Consortium (FICC) database. The FICC database is a nationwide database including all ICU-treated patients from the majority of all Finnish ICUs [34]. In Finland, all specialized tertiary intensive care of patients with TBI is centralized to five university hospital ICUs. Four of these ICUs, covering approximately two-thirds of the population in Finland, participate in the FICC. From these four tertiary ICUs, we included all adult TBI patients (age ≥ 18 years) admitted between January 1, 2003, and December 31, 2013 (readmissions excluded). TBI patients were identified by Acute Physiology and Chronic Health Evaluation (APACHE) III diagnostic codes and the diagnoses were manually verified by screening health records and reviewing primary head computer tomography (CT) scans [31]. Patients were excluded, if no CT was available, Glasgow Coma Scale (GCS) or pre-admission functional status was missing.

### Definition of covariates

ICU-related variables were retrieved from the FICC database. The GCS score is defined according to the APACHE II definition as the worst measured GCS score during the first ICU day [20]. For intubated and/or sedated patients, the last reliable GCS score preceding sedation is used. The FICC uses a modified version of the World Health Organization/Eastern Cooperative Oncology Group (WHO/ECOG) classification for pre-admission functional status (fit for work or equal, unfit for work but independent in self-care, partially dependent in self-care, totally dependent in self-care) [27]. We classified all admission CT scans according to the Marshall CT classification [25]. We defined a significant chronic comorbidity according to the APACHE II and Simplified Acute Physiology Score (SAPS) II [20, 23]. We defined Intracranial Pressure (ICP) monitoring through the Therapeutic Intervention Scoring System (TISS) 76 that is routinely collected for the FICC database [19]. We used the NOMESCO Classification of Surgical Procedures Finland (NCSP-F) for the definition of external ventricular drain (EVD, NCSP-F code AAF00), craniotomy for hematoma evacuation (NCSP-F code AAD00, AAD05, AAD15) and for decompressive craniectomy (NCSP-F code AAK80).

### Psychotropic medication purchases

In Finland, patients get physician-prescribed medication, including psychiatric medication, reimbursed by the Social Insurance Institution with a maximum out-of-pocket payment of roughly 600 euros per calendar year. After the out-of-pocket ceiling is reached, patients pay 2.50 euros per medication and per purchase regardless of the cost.

We obtained data on purchased psychotropic medication from the Social Insurance Institution from January 1, 2003, to December 31, 2013. We defined psychotropic medication as an Anatomical Therapeutic Chemical (ATC) classification system code of N05A-C* (N05A = antipsychotics, N05B = anxiolytics, N05C = hypnotics and sedatives) and N06A-C* (N06A = antidepressants, N06B = psychostimulants, N06C = psycholeptics and psychoanaleptics in combination, excluding N06D*, anti-dementia drugs) [40]. We considered psychotropic medication use if the patient purchased the medication at least two times. We defined start of psychotropic medication use at the date of first purchase. We separately classified according to pre-TBI use and post-TBI use.

### Statistical methods

We used SPSS Statistics 25.0 for mac OS (IBM Corp, Armonk, NY) and Stata Statistical Software for macOS (StataCorp LP, College Station, TX) for the statistical analyses.

We compared categorical data between groups using a two-sided *χ*^2^ test. We tested continuous data for skewness. We present normally distributed data as means with standard deviations (SD) and non-parametric data as medians with interquartile range (IQR). We compared normally distributed data between groups using a *t*-test and non-parametric data using a Mann–Whitney *U* test.

We tested the association between risk factors and the odds of use of psychotropic medication using multivariable logistic regression models. In the logistic regression models, we adjusted for age, gender, GCS score, chronic comorbidity, WHO/ECOG classification, Marshall CT classification and a modified SAPS II (excluding age, GCS score and chronic comorbidity) [31].

We tested the association between pre-TBI psychotropic medication use and 1-year mortality and between post-TBI psychotropic medication use and late mortality (in 1-year survivors) using logistic regression analysis, adjusting for the aforementioned factors. We defined late mortality as all-cause mortality that happened later than 1 year after TBI, as most of the death attributable to TBI occurs in the first year [1].

## Results

**Fig. 1:**
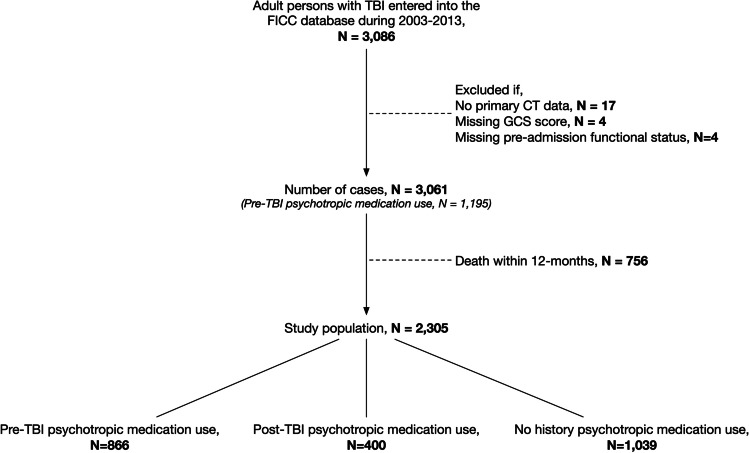
Flow chart. *Abbreviations: TBI, traumatic brain injury; FICC, Finnish Intensive Care Consortium; CT, computerized tomography; GCS, Glasgow Coma Scale*

Of 3061 patients, 1195 (39%) had a history of psychotropic medication use prior to the TBI (Fig. 1). Of the 3061 patients, 2305 (75%) were alive 1 year after the TBI. Of these, 866 patients (38%) had a history of psychotropic medication use prior to TBI, 400 patients (17% of all survivors) were prescribed a psychotropic medication after TBI and 1039 patients (45%) had no prior or new history of psychotropic medication use.

Median time from first psychotropic medication purchase to TBI was 4.0 years (IQR 2.0–6.4) for those with a history of psychotropic medication use prior to TBI. Median time from TBI to first psychotropic medication purchase was 0.6 years for those without a history of prior use (IQR 0.2–1.5 years).

Patients with a history of pre-TBI use of psychotropic medication (*N* = 1195) had been less frequently fit for work (51% vs. 68%), were more often female (29% vs. 19%) and had more severe chronic comorbidities (11% vs. 7%) than patients without a pre-TBI history of psychotropic medication use (Table 1).

**Table 1:** Baseline characteristics and treatment of traumatic brain injury patients

Variables	All patients (*N* = 3061)	Pre-TBI psychotropic medication use (*N* = 1195)	No pre-TBI psychotropic medication use (*N* = 1866)	*p*-value
Age, median (IQR)	56 (41, 67)	57 (45, 68)	54 (38, 66)	< 0.001
18–40 years	745 (24%)	226 (19%)	519 (28%)	< 0.001
41–64 years	1434 (47%)	611 (51%)	823 (44%)	
≥ 65 years	882 (29%)	358 (30%)	524 (28%)	
GCS score, median (IQR)	9 (5, 14)	9 (5, 14)	9 (5, 14)	0.92
3–8	1443 (47%)	557 (47%)	886 (47%)	0.89
9–12	596 (19%)	236 (20%)	360 (19%)	
13–15	1022 (33%)	402 (34%)	620 (33%)	
Females	701 (23%)	348 (29%)	353 (19%)	< 0.001
Pre-admission performance status*				
Fit for work or equal	1872 (61%)	611 (51%)	1261 (68%)	< 0.001
Unfit for work, but independent in self-care	957 (31%)	451 (38%)	506 (27%)	
Partially dependent in self-care	178 (6%)	99 (8%)	79 (4%)	
Totally dependent in self-care	54 (2%)	34 (3%)	20 (1%)	
Significant chronic comorbidity†	261 (9%)	136 (11%)	125 (7%)	< 0.001
SAPS II score, median (IQR)	35 (23, 50)	35 (25, 50)	35 (23, 49)	0.059
Marshall CT classification				
DI I	317 (10%)	114 (10%)	203 (11%)	< 0.001
DI II	988 (32%)	350 (29%)	638 (34%)	
DI III	287 (9%)	107 (9%)	180 (10%)	
DI IV	45 (1%)	11 (1%)	34 (2%)	
EML V/NEML VI	1424 (47%)	613 (51%)	811 (43%)	
Craniotomy and hematoma evacuation	1220 (40%)	532 (45%)	688 (37%)	< 0.001
Decompressive craniectomy	50 (2%)	14 (1%)	36 (2%)	0.11
External ventricular drain	156 (5%)	56 (5%)	100 (5%)	0.41
ICP monitoring	724 (24%)	248 (21%)	476 (26%)	0.003
Mechanical ventilation	2042 (67%)	780 (65%)	1262 (68%)	0.18
LOS ICU, days, median (IQR)	2 (1, 4)	2 (1, 3)	2 (1, 5)	0.005
LOS hospital, days, median (IQR)	6 (3, 11)	5 (3, 19)	6 (3, 11)	0.0012

^*^A modified World Health Organization/Eastern Cooperative Oncology Group classification system implemented by the Finnish Intensive Care Consortium

^†^Any chronic comorbidity according to APACHE II or to SAPS II

*Abbreviations: APACHE*, Acute Physiology and Chronic Health Evaluation; *DI*, diffuse injury; *GCS*; Glasgow Coma Scale; *EML/NEML*, evacuated/non-evacuated mass lesion; *LOS*, length of stay; *ICP*, intracranial pressure; *ICU*, intensive care unit; *SAPS*, Simplified Acute Physiology Score; *TBI*, traumatic brain injury

One-year survivors with a new post-TBI psychotropic medication use had lower GCS scores (median 9 vs. 12), had been less frequent fit for work (67% vs. 76%), more often underwent craniotomy for mass lesion (42% vs. 31%), were more often mechanically ventilated (73% vs. 56%), had higher SAPS II scores (34 vs. 28) and more often a mass lesion on the admission head CT scan (41% vs. 35%) than 1-year survivors without a need for psychotropic medication (Table 2).

**Table 2:** Baseline characteristics and treatment of 1-year survivors of traumatic brain injury patients who had no pre-injury history of psychotropic drug use

Variables	All patients without pre-TBI drug use (*N* = 1439)	Post-TBI psychotropic medication use (*N* = 400)	No psychotropic medication use (*N* = 1039)	*p*-value
Age, median (IQR)	51 (33, 63)	53 (36, 64)	51 (31, 63)	0.08
18–40 years	472 (33%)	118 (30%)	354 (34%)	0.25
41–64 years	639 (44%)	185 (46%)	454 (44%)	
≥ 65 years	328 (23%)	97 (24%)	231 (22%)	
GCS score, median (IQR)	11 (6, 14)	9 (5–13)	12 (7–14)	< 0.001
3–8	542 (38%)	191 (48%)	351 (34%)	< 0.001
9–12	309 (21%)	91 (23%)	218 (21%)	
13–15	588 (41%)	118 (30%)	470 (45%)	
Females	266 (18%)	84 (21%)	182 (18%)	0.13
Pre-admission performance status*				
Fit for work or equal	1058 (74%)	270 (68%)	788 (76%)	0.003
Unfit for work, but independent in self-care	330 (23%)	118 (30%)	212 (20%)	
Partially dependent in self-care	41 (3%)	9 (2%)	32 (3%)	
Totally dependent in self-care	10 (1%)	3 (1%)	7 (1%)	
Significant chronic comorbidity†	70 (5%)	16 (4%)	54 (5%)	0.34
SAPS II score, median (IQR)	30 (20, 41)	34 (24, 45)	28 (20, 40)	< 0.001
Marshall CT classification				
DI I	179 (12%)	35 (9%)	144 (14%)	0.001
DI II	587 (41%)	156 (39%)	431 (41%)	
DI III	126 (9%)	35 (9%)	91 (9%)	
DI IV	18 (1%)	11 (3%)	7 (1%)	
EML V/NEML VI	529 (37%)	163 (41%)	366 (35%)	
Craniotomy and hematoma evacuation	487 (34%)	169 (42%)	318 (31%)	< 0.001
Decompressive craniectomy	29 (2%)	8 (2%)	22 (2%)	0.66
External ventricular drain	79 (5%)	27 (7%)	52 (5%)	0.19
ICP monitoring	336 (23%)	114 (29%)	222 (21%)	0.004
Mechanical ventilation	869 (60%)	290 (73%)	579 (56%)	< 0.001
LOS ICU, days, median (IQR)	2 (1, 5)	2 (1, 6)	2 (1, 4)	< 0.001
LOS hospital, days, median (IQR)	7 (4, 12)	8 (4–15)	6 (4, 11)	< 0.001

Analyses do not include those with a history of psychotropic medication use prior to the index hospitalization

^*^A modified World Health Organization/Eastern Cooperative Oncology Group classification system implemented by the Finnish Intensive Care Consortium

^†^Any chronic comorbidity according to APACHE II or to SAPS II

*Abbreviations: APACHE*, Acute Physiology and Chronic Health Evaluation; *DI*, diffuse injury; *GCS*; Glasgow Coma Scale; *EML/NEML*, evacuated/non-evacuated mass lesion; *LOS*, length of stay; *ICP*, intracranial pressure; *ICU*, intensive care unit; *SAPS*, Simplified Acute Physiology Score; *TBI*, traumatic brain injury

In patients with pre-TBI psychotropic medication use, the most common psychotropic drugs were antidepressants (70%), hypnotics and sedatives (58%) and anxiolytics (47%). Among the 1-year survivors with new use of psychotropic medication, antidepressants (61%), antipsychotics (35%) and anxiolytics (26%) were the most frequently used psychotropic medications. Furthermore, 27% used multiple psychotropic medications (Supplemental Table 1).

### Risk factors for psychotropic medication

Among the 1439 1-year survivors without a history of pre-TBI psychotropic medication use, a higher GCS score was associated with lower odds (OR 0.93 per point, 95% CI 0.90–0.96) and a diffuse injury IV was associated with higher odds (OR 3.37, 95% CI 1.27–9.01, using Marshall DI II as the reference) of psychotropic medication use (Table 3). In a similar model, using GCS score as a categorical variable and GCS 13–15 as the reference, GCS of 3–8 had an OR of 1.92 (95% CI 1.42–2.59) and GCS 9–12 had an OR of 1.46 (95% CI 1.05–2.04).

**Table 3:** Multivariable logistic regression model showing association between patient demographics, markers of traumatic brain injury severity and psychotropic medication use in 1-year survivors

Variable	Odds ratio (95% CI)	*p*-value
Age	1.01 (0.99–1.02)	0.130
Sex		
Male	1.0	
Female	1.31 (0.97–1.77)	0.072
GCS score	0.93 (0.90–0.96)	< 0.001
Significant comorbidity	0.68 (0.38–1.22)	0.196
Pre-admission functional status		
Independent in ADL	1.0	
Dependent in ADL	0.59 (0.30–1.17)	0.135
Modified SAPS II score	1.02 (1.00–1.04)	0.101
Marshall CT class		
DI I	0.71 (0.46–1.08)	0.108
DI II	1.0	
DI III	0.91 (0.58–1.41)	0.672
DI IV	3.37 (1.27–9.01)	0.015
EML V/NEML VI	1.07 (0.81–1.41)	0.635

*Abbreviations*: *ADL*, activities of daily living; *CI*, confidence interval; *DI*, diffuse injury; *EML*, evacuated mass lesion; *GCS*, Glasgow Coma Scale; *NEML*, non-evacuated mass lesion; *SAPS*, Simplified Acute Physiology Score

After adjusting for TBI severity, an association between craniotomy for hematoma evacuation (OR 1.51, 95% CI 1.08–2.12) and post-TBI psychotropic medication use was found. No association between ICP monitoring, EVD or decompressive craniectomy and post-TBI use of psychotropics was found (Supplemental Table 2).

### Psychotropic medication and mortality

Of 2305 1-year survivors, 547 patients (24%) died during follow-up. Median follow-up time was 6.4 years (IQR 4.3–9.3). New psychotropic medication use (OR 1.60, 95% CI 1.19–2.17) and a history of pre-TBI psychotropic medication use (OR 1.82, 95% CI 1.19–2.17) were associated with increased odds of late-mortality in 1-year survivors (Table 4).

**Table 4:** Multivariable logistic regression model showing association between use of psychotropic medication and late mortality in 1-year survivors

Variable	Odds ratio (95% CI)	*p*-value
Age	1.05 (1.04–1.05)	< 0.001
Sex		
Male	1.0	
Female	0.58 (0.45–0.76)	< 0.001
GCS score	0.98 (0.95–1.01)	0.172
Significant comorbidity	1.68 (1.15–2.45)	0.008
Pre-admission functional status		
Independent in ADL	1.0	
Dependent in ADL	2.70 (1.76–4.12)	< 0.001
Modified SAPS II score	1.01 (0.99–1.02)	0.530
Marshall CT class		
DI I	0.82 (0.53–1.27)	0.370
DI II	1.0	
DI III	0.99 (0.64–1.53)	0.953
DI IV	1.10 (0.39–3.10)	0.861
EML V/NEML VI	1.72 (1.35–2.19)	< 0.001
Psychotropic medication use		
No	1.0	
Pre-TBI use	1.82 (1.43–2.30)	< 0.001
New post-TBI use	1.60 (1.19–2.17)	0.002

Median follow-up time was 6.4 years (interquartile range 4.3–9.3)

*Abbreviations*: *ADL*, activities of daily living; *CI*, confidence interval; *DI*, diffuse injury; *EML*, evacuated mass lesion; *GCS*, Glasgow Coma Scale; *NEML*, non-evacuated mass lesion; *SAPS*, Simplified Acute Physiology Score

Including all patients, pre-TBI use of psychotropic medication was not associated with increased odds of 1-year mortality (Supplemental Table 3).

## Discussion

### Key findings

In this retrospective multi-centre study, we found that the use of psychotropic medication is common both in patients experiencing TBI and among survivors. Four out of ten patients with TBI used psychotropic medications pre-injury. Among the long-term survivors, three out of ten patients without a history of prior psychotropic medication use were prescribed a psychotropic medication. These figures are notably higher than the use of psychotropic medication (18%) in the whole adult population in Finland in 2013 [40]. Thus, patients using psychotropic medication are strongly overrepresented among ICU-treated patients with TBI. New post-TBI psychotropic medication use was associated with more severe TBI and appeared especially common in patients needing craniotomy for hematoma evacuation. Importantly, new psychotropic medication use after TBI was associated with an increased risk for late mortality.

Few studies have reported the prevalence of pre- and post-TBI use of psychotropic drugs [13, 21]. However, several studies have shown the abundance of psychiatric sequelae and illness among TBI survivors: prevalence of 14–77% for depression, 2–17% for bipolar disorder, 3–28% for generalized anxiety disorders and 5–28% for substance use have been reported [5, 10, 15, 22, 32, 33, 39]. Of patients with ICU-treated severe TBI, approximately 30% suffer from major depressive disorders and 33% from major personality change [6]. In comparison, the prevalence of psychiatric symptoms in general ICU survivors seems to range between 17 and 44% [26, 28, 30, 38]. Thus, the high prevalence of the use of psychotropic medications in the current study is not surprising.

### Clinical phenotypes increasing the odds of psychotropic prescription

Previous studies have found that the severity of TBI correlates with the incidence of psychiatric illnesses [9]. Studies on patients with mild TBI have generally found lower incidences of depression than studies investigating moderate-to-severe TBI patients [10, 32, 33]. On the contrary, Jorge et al. [17] did not find an association between the incidence of depression and severity of TBI. It has been speculated that a more severe TBI and its related post-traumatic amnesia may have a protective role for post-traumatic stress disorder (PTSD) and, thus, the relationship between TBI severity and depression is not linear [33, 37]. Furthermore, it has been proposed that the brain injury area location may be more important than general markers of TBI severity such as initial GCS or duration of coma in terms of probability of depression and other psychiatric disorders [8, 17, 33, 37]. Specific locations of lesions in different psychiatric disorders have been found as follows: depression, lower bilateral hippocampal volume [16]; mania, lesions in temporal basal poles [15]; obsessive-compulsory disorder, orbitofrontal cortex, cortex cinguli, nucleus caudatus [3]; psychosis, damage in frontal and orbital lobes [11, 36]; alcohol-related disorders, prefrontal cortex volume reduction [18]. These are in-line with our observations that the more diffuse injuries (Marshall CT class IV) can also damage the parts of the brain described earlier, thus specifically causing organic psychiatric disorders.

The Marshall CT classification has not been widely used to classify radiological findings in previous studies dealing with TBI and psychiatric disorders. In the study by Diaz et al., Marshall CT classification or presence of traumatic subarachnoid haemorrhage (SAH) did not associate with post-TBI psychiatric disorder after severe TBI [6]. We found that Marshall CT class IV associated with increased odds of new psychotropic medication use after TBI. This may be interpreted as an association between extensive diffuse brain injury and psychiatric sequela.

Interestingly, we found that craniotomy and hematoma evacuation associated with increased odds of psychotropic medication use, but we did not find a comparable association of the combined Marshall classes V and VI and later medication use. Considering that the cut off > 25 cm^3^ for the Marshall classes V and VI is purely radiological and arbitrary, it is not that surprising. For example, the variable craniotomy and hematoma evacuation indirectly represents a combination of radiological mass effect, lowered GCS and reasonable patient prognosis. Thus, the craniotomy variable captures more information than the Marshall classification and might be considered as an indicator of a more severe brain injury. However, this finding should be interpreted with caution as it may be affected by several unmeasured factors.

New psychotropic medication use in 1-year survivors was associated with increased odds of late mortality, even after adjusting for TBI severity. Other risk factors for the need of psychotropic medication after TBI are, for example, unfavourable Glasgow Outcome Scale (GOS) [5], history of previous head injury [5], history of psychiatric illness [5, 33], unemployment before TBI [7, 17] and lower levels of education [7]. Thus, psychiatric sequelae after TBI are multifactorial seems to increase risk of late mortality. Early identification of psychiatric comorbidity in TBI survivors may be important to individualize and promote favourable functional and psychosocial recovery.

The very high proportion of patients with TBI combined with a history of psychotropic medication use, the high proportion of new users among TBI survivors and the increased risk of late mortality highlight the need for multi-professional collaboration between fields of neurosurgery, neurology and psychiatrics during the follow-up and rehabilitation of survivors of a TBI. Early identification of potential psychiatric burden and early-stage psychiatric evaluation as the part of multidisciplinary approach in TBI patients might overall influence the final outcomes and necessity of psychiatric medicine initiation and/or psychotherapeutic late interventions, which would require further and more specific investigation. Whether these measures by a multi-professional team could improve outcomes and should be studied.

### Strengths and limitations

Our study has numerous strengths. It is a multi-centre observational study including four out of the five Finnish tertiary ICUs treating TBI patients. This means that our study includes the majority of TBI patients requiring ICU treatment in Finland. The referral population of the four neurointensive ICU’s is approximately 3.5 million people, encompassing two-thirds of the Finnish population. We managed to do complete follow-up of the patient medication history as Finland has a government backed tax-funded system for drug reimbursement. Virtually all reimbursed prescriptions are entered into the system. Furthermore, the reimbursement decreases the price of drugs that could otherwise prevent patients of the treatment.

A limitation of the study is that we cannot exclude the possibility of off-label prescription of psychotropic medications, which might lead to positive bias in our study. However, negative bias might arise due to the medicine-free treatment (i.e. psychotherapeutic and/or supportive intervention only) of certain psychiatric conditions. Also, we acknowledge the cultural and social influences as a potential factor affecting the threshold for seeking the help of psychiatrist, and thus influencing the compliance of psychotropic drugs use. It should also be highlighted that we used psychotropic medication as a proxy for psychiatric comorbidity. We did not identify specific locations of cerebral contusions. Thus, we could not consider the specific lesions, e.g. damage to frontal and orbital lobes earlier related to psychosis after TBI [11, 36]. Also, we could not assess the association between pre-trauma socioeconomical factors and risk of psychiatric sequel. The causes of death were not available and, thus, reasons for the increased late mortality in psychotropic medication users remain unclear.

## Conclusions

The use of psychotropic medication in ICU-treated patients with TBI is common. Four out of ten patients had a history of psychotropic medication use prior to the TBI, and among survivors without prior use of psychotropic medication, three out of ten were later prescribed a new medication. A lower GCS score and a diffuse brain injury with midline shift were independently associated with increased odds of future psychotropic medication use. New psychotropic medication use in survivors was associated with increased odds of late mortality. Additional studies are required to define whether early identification and treatment of psychiatric sequelae after TBI could result in more favourable outcomes.

## Supplementary Information

Below is the link to the electronic supplementary material.

Supplementary file1 (PDF 104 KB)

Supplementary file2 (PDF 36 KB)

Supplementary file3 (PDF 97 KB)
