# Supplementary material for: Psychotropic medication use among patients with a traumatic brain injury treated in the intensive care unit: a multi-centre observational study
Source: Acta Neurochir (Wien). 2021 Aug 11;163(10):2909–17. doi: 10.1007/s00701-021-04956-3 (PMC8437905; doi:10.1007/s00701-021-04956-3)
Supplement: Supplementary file 1 — Supplementary file1 (PDF 104 KB) [file 701_2021_4956_MOESM1_ESM.pdf]

Psychotropic medication use among patients with a traumatic brain injury treated in the intensive care unit: a multi-centre observational study

Acta Neurochirurgica

Juho Vehviläinen*, MD, MSc [1], Markus B. Skrifvars, MD, PhD [2], Matti Reinikainen, MD, PhD [3], Stepani Bendel, MD, PhD [3], Ivan Marinkovic, MD, PhD [4], Tero Ala-Kokko, MD, PhD [5], Sanna Hoppu, MD, PhD [6], Ruut Laitio, MD, PhD [7], Jari Siironen, MD, PhD, [1] Rahul Raj, MD, PhD [1]

1. Department of Neurosurgery, Helsinki University Hospital and University of Helsinki, Helsinki, Finland
2. Department of Emergency Care and Services, University of Helsinki and Helsinki University Hospital, Helsinki, Finland.
3. Department of Intensive Care, Kuopio University Hospital & University of Eastern Finland, Kuopio, Finland
4. Department of Neurology, Helsinki University Hospital and University of Helsinki, Helsinki, Finland
5. Department of Intensive Care, Oulu University Hospital & University of Oulu, Oulu, Finland
6. Department of Intensive Care and Emergency Medicine Services, Tampere University Hospital & University of Tampere, Tampere, Finland
7. Department of Intensive Care, Turku University Hospital & University of Turku, Turku, Finland

Corresponding author*: **Juho Vehviläinen**

- E-mail: juho.vehvilainen@helsinki.fi

Supplemental Table 1: Psychotropic medication use broken down by different Anatomical Therapeutic Chemical (ATC) classes. We considered psychotropic medication use if the patient purchased the medication ≥2 times. The number of patients using psychotropic drugs is broken down into four groups: All one-year survived TBI patients in the study, patients who had pre-TBI psychotropic use, and patients who were prescribed psychotropics post-TBI, all-patients with TBI and pre-TBI psychotropic medication use. The psychotropic use in general Finnish adult population is in the last column. N = number of patients

**Variables**	**One-year survivors**			**All patients**	**Use in general adult population in 2013***
	**All TBI patients (N=2,305)**	**Pre-TBI users (N=866)**	**New post-TBI users (N=400)**	**Pre-TBI users (N=1,195)**	
ATC classes	N (%)	N (%)	N (%)	N (%)	
**N05 Psycholeptics**	1,016 (44%)	760 (88%)	256 (64%)	1,037 (87%)	11.7%
Multiple N05 drugs	469 (20%)	393 (45%)	76 (19%)	491 (41%)	No data
N05A Antipsychotics	486 (21%)	347 (40%)	139 (35%)	420 (35%)	3.9%
N05B Anxiolytics	531 (23%)	426 (49%)	105 (26%)	560 (47%)	4.0%
N05C Hypnotics and sedatives	596 (26%)	505 (58%)	91 (23%)	690 (58%)	6.4%
**N06 Psychoanaleptics**	911 (40%)	659 (76%)	252 (63%)	859 (72%)	11.1%
Multiple N06 drugs	46 (2.0%)	41 (4.7%)	5 (1.2%)	51 (4.3%)	No data
N06A Antidepressants	889 (39%)	645 (74%)	244 (61%)	836 (70%)	9.6%
N06B Psychostimulants, Agents used for ADHD, and Nootropics	23 (1.0%)	19 (2.2%)	4 (1.0%)	20 (1.7%)	0.1%
N06C Psycholeptics and Psychoanaleptics in combination	38 (1.7%)	32 (3.7%)	6 (1.5%)	49 (4.1%)	0.4%
**Multiple drug use N05 and N06 classes**	661 (29%)	553 (64%)	108 (27%)	701 (59%)	No data
**Total (N05 and/or N06)**	1,266 (55%)	866	400	1,195	18%

*Based upon data from Kela (the Social Insurance Institution of Finland)
