# Supplementary material for: Psychotropic medication use among patients with a traumatic brain injury treated in the intensive care unit: a multi-centre observational study
Source: Acta Neurochir (Wien). 2021 Aug 11;163(10):2909–17. doi: 10.1007/s00701-021-04956-3 (PMC8437905; doi:10.1007/s00701-021-04956-3)
Supplement: Supplementary file 2 — Supplementary file2 (PDF 36 KB) [file 701_2021_4956_MOESM2_ESM.pdf]

Psychotropic medication use among patients with a traumatic brain injury treated in the intensive care unit: a multi-centre observational study

Acta Neurochirurgica

Juho Vehviläinen*, MD, MSc [1], Markus B. Skrifvars, MD, PhD [2], Matti Reinikainen, MD, PhD [3], Stepani Bendel, MD, PhD [3], Ivan Marinkovic, MD, PhD [4], Tero Ala-Kokko, MD, PhD [5], Sanna Hoppu, MD, PhD [6], Ruut Laitio, MD, PhD [7], Jari Siironen, MD, PhD, [1] Rahul Raj, MD, PhD [1]

1. Department of Neurosurgery, Helsinki University Hospital and University of Helsinki, Helsinki, Finland
2. Department of Emergency Care and Services, University of Helsinki and Helsinki University Hospital, Helsinki, Finland.
3. Department of Intensive Care, Kuopio University Hospital & University of Eastern Finland, Kuopio, Finland
4. Department of Neurology, Helsinki University Hospital and University of Helsinki, Helsinki, Finland
5. Department of Intensive Care, Oulu University Hospital & University of Oulu, Oulu, Finland
6. Department of Intensive Care and Emergency Medicine Services, Tampere University Hospital & University of Tampere, Tampere, Finland
7. Department of Intensive Care, Turku University Hospital & University of Turku, Turku, Finland

Corresponding author*: **Juho Vehviläinen**

- E-mail: juho.vehvilainen@helsinki.fi

Supplemental Table 2: Multivariable logistic regression model showing association between interventions and psychotropic medication use in 12-month survivors after adjusting for TBI severity

Variable	Odds ratio (95% CI)	p-Value
**Intracranial pressure monitor**	1.04 (0.75-1.47)	0.800
**External ventricular drain**	1.02 (0.59-1.74)	0.952
**Craniotomy for hematoma evacuation**	1.51 (1.08-2.12)	0.016
**Decompressive craniectomy**	0.63 (0.25-1.55)	0.311

Multivariable logistic regression adjusting for the independent variables: age, sex, GCS score, significant comorbidity, pre-admission functional status, Marshall CT class, modified SAPS II score.

Abbreviations: CI, Confidence Interval; GCS, Glasgow Coma Scale; SAPS, Simplified Acute Physiology Score
