# Supplementary material for: Psychotropic medication use among patients with a traumatic brain injury treated in the intensive care unit: a multi-centre observational study
Source: Acta Neurochir (Wien). 2021 Aug 11;163(10):2909–17. doi: 10.1007/s00701-021-04956-3 (PMC8437905; doi:10.1007/s00701-021-04956-3)
Supplement: Supplementary file 3 — Supplementary file3 (PDF 97 KB) [file 701_2021_4956_MOESM3_ESM.pdf]

Psychotropic medication use among patients with a traumatic brain injury treated in the intensive care unit: a multi-centre observational study

Acta Neurochirurgica

Juho Vehviläinen*, MD, MSc [1], Markus B. Skrifvars, MD, PhD [2], Matti Reinikainen, MD, PhD [3], Stepani Bendel, MD, PhD [3], Ivan Marinkovic, MD, PhD [4], Tero Ala-Kokko, MD, PhD [5], Sanna Hoppu, MD, PhD [6], Ruut Laitio, MD, PhD [7], Jari Siironen, MD, PhD, [1] Rahul Raj, MD, PhD [1]

1. Department of Neurosurgery, Helsinki University Hospital and University of Helsinki, Helsinki, Finland
2. Department of Emergency Care and Services, University of Helsinki and Helsinki University Hospital, Helsinki, Finland.
3. Department of Intensive Care, Kuopio University Hospital & University of Eastern Finland, Kuopio, Finland
4. Department of Neurology, Helsinki University Hospital and University of Helsinki, Helsinki, Finland
5. Department of Intensive Care, Oulu University Hospital & University of Oulu, Oulu, Finland
6. Department of Intensive Care and Emergency Medicine Services, Tampere University Hospital & University of Tampere, Tampere, Finland
7. Department of Intensive Care, Turku University Hospital & University of Turku, Turku, Finland

Corresponding author*: **Juho Vehviläinen**

- E-mail: juho.vehvilainen@helsinki.fi

Supplemental Table 3: Logistic regression model showing the association between pre-TBI use of any psychotropic medication and risk of one-year mortality

Variable	Odds ratio (95% CI)	p-Value
**Age**	1.04 (1.04–1.05)	<0.001
**Gender**		
Male	1.0	
Female	0.79 (0.63–1.01)	0.061
**Significant comorbidity**	1.53 (1.11–2.13)	0.010
**Pre-admission functional status**		
Independent in ADL	1.0	
Dependent in ADL	1.90 (1.36–2.67)	<0.001
**Modified SAPS II score**	1.08 (1.06–1.10)	<0.001
**Marshall CT class**		
DI I	1.30 (0.81–2.08)	0.274
DI II	1.0	
DI III	2.55 (1.73–3.74)	<0.001
DI IV	3.39 (1.61–7.17)	0.0001
EML/NEML	2.12 (1.63–2.76)	<0.001
**Pre-TBI use of any psychotropic medication**	1.18 (0.96–1.46)	0.113

Abbreviations: ADL, Activities of Daily Living; CI, Confidence Interval; DI, Diffuse Injury; GCS, Glasgow Coma Scale; SAPS, Simplified Acute Physiology Score
